# Successful thrombolytic therapy is associated with increased granulocyte CD15 expression and reduced stroke‐induced immunosuppression

**DOI:** 10.1002/brb3.2732

**Published:** 2022-09-16

**Authors:** Katalin Anna Béres‐Molnár, Ágnes Czeti, Ferenc Takács, Gábor Barna, Dániel Kis, Gabriella Róka, András Folyovich, Gergely Toldi

**Affiliations:** ^1^ Department of Neurology and Stroke Szent János Hospital Budapest Hungary; ^2^ 1st Department of Pathology and Experimental Cancer Research Semmelweis University Budapest Hungary; ^3^ Department of Laboratory Medicine Semmelweis University Budapest Hungary; ^4^ Liggins Institute University of Auckland Auckland New Zealand

**Keywords:** acute ischemic stroke, CD64, inflammation, monocyte

## Abstract

**Objectives:**

Stroke‐induced immunosuppression (SIIS) increases the risk of poststroke infections. We aimed to determine whether failed versus successful thrombolytic therapy (TT) resulted in SIIS‐associated changes in peripheral granulocyte markers at 1 week following the insult.

**Methods:**

We collected peripheral blood samples from 19 patients with acute ischemic stroke undergoing TT within 6 h after the onset of their first symptoms and 7 days after the insult. Age‐matched controls were sampled on one occasion. We compared the expression of CD15 and CD64 on monocytes, granulocytes, and lymphocytes using flow cytometry.

**Results:**

The proportion of granulocytes and CD15+ granulocytes was comparable between controls and stroke patients at both time points. While the proportion of CD15bright granulocytes was also comparable, the mean fluorescence intensity (MFI) of CD15 on this subset was reduced in stroke patients by day 7 but was overall higher at both time points compared to controls. The MFI of CD15 on granulocytes was lower in stroke patients with failed TT than in those with successful TT 1 week after the insult.

**Conclusions:**

Our current results indicate that TT may not only acutely reduce the systemic inflammatory response following stroke but may also play a role in reversing SIIS at a later stage following the insult, as reflected by the higher expression of the CD15 marker on granulocytes following successful TT.

## Graphical abstract

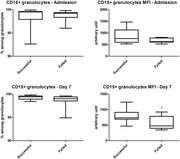

## 1 INTRODUCTION

Stroke‐induced infections, such as stroke‐associated pneumonia, represent one of the most common poststroke complications increasing mortality rates. Nearly one‐third of stroke patients develop infection, worsening their functional outcome (Westendorp et al., 2011).

The immunological mechanism increasing the susceptibility of stroke patients to infection is termed stroke‐induced immunosuppression (SIIS) and has received considerable interest in recent years (Faura et al., 2021). Understanding the immunological response and changes to the peripheral immune system that evolve after stroke is of key importance to improve complication rates and outcomes via early diagnosis and appropriate therapeutic interventions.

In our earlier study, we investigated the relevance of soluble and cellular inflammatory markers in SIIS in the first week following the insult in acute ischemic stroke (AIS) patients who did not develop poststroke infection. We identified that the prevalence of CD64+ neutrophils may reflect a biphasic alteration of the peripheral immune response following AIS, increasing within 6 h after the insult compared to controls but decreasing below baseline by 1 week after the insult in the absence of infection (Folyovich et al., 2014). CD64, or Fc gamma receptor I, is upregulated on neutrophil granulocytes upon activation and is increasingly recognized as a diagnostic marker of sepsis superior to C‐reactive protein or hematological markers. Its upregulation is mediated by proinflammatory cytokines, such as interferon gamma (IFN‐g) and granulocyte colony stimulating factor (G‐CSF), which are produced in large quantities in response to infection or tissue injury. CD64 plays a role in antigen capture, phagocytosis of IgG/antigen complexes, and antibody‐dependent cellular cytotoxicity (Hoffmann, 2009).

In addition to CD64, CD15 is another important marker of granulocyte function. CD15 is a carbohydrate antigen associated with glycoproteins and glycolipids (Nakayama et al., 2001). An important mechanism of CD15 expression is the induction of alpha (2‐3)‐sialidase activity, which yields CD15 from cell‐surface sialyl‐CD15 (CD15s). This differentiation‐associated conversion of CD15s to CD15 occurs mostly on glycoproteins. In addition to monocytes and promyelocytes, it is highly expressed by neutrophils and plays a role in various neutrophil functions, including adhesion to dendritic cells, phagocytosis, degranulation, and respiratory bursts (Melnick et al., 1985; Stocks et al., 1990).

Thrombolytic therapy (TT) is an effective therapeutic intervention to reduce complications and disability arising from AIS. However, as the therapeutic time window is narrow and patients need to meet specific criteria to maximize efficiency, not all patients qualify for this therapy (Berge et al., 2021; Powers et al., 2019). Furthermore, TT is known to reduce the systemic inflammatory response following stroke (Audebert et al., 2004; Ye et al., 2015). Therefore, we hypothesized that successful TT could also influence the development of SIIS.

In the present study, we aimed to determine whether failed versus successful TT resulted in SIIS‐associated changes in peripheral granulocyte markers at 1 week following the insult. We compared the expression of the CD15 and CD64 markers in AIS patients and age‐matched controls. The patient group was further divided according to the success of TT.

## 2 MATERIALS AND METHODS

Study participants were enrolled at the Department of Neurology of Szent János Hospital, Budapest, Hungary. Peripheral blood samples were collected from 19 patients diagnosed with AIS upon admission, within 6 h after the onset of their first symptoms. A repeat peripheral blood sample was collected from each patient 7 days after the insult. The severity of neurological deficit was assessed using the NIHSS and modified Rankin scores upon admission and discharge. TT was defined as successful if no ischemic lesion was present on the repeat cranial computed tomography following thrombolysis, and the modified Rankin score at discharge was not worse than upon admission. TT was performed in line with the guidelines of the European Stroke Organization (2008, revised in 2021) (Berge et al., 2021). Clinical characteristics of patients are summarized in Table 1.

**TABLE 1:** Clinical characteristics of acute ischemic stroke patients enrolled in the study

	Successful TT (*n* = 11)	Failed TT (*n* = 8)
Age (years)	76 [74.5–84.5]	80.5 [77–82]
Gender (male/female)	4/7	3/5
NIHSS score at admission	5 [4–8]	6.5 [5–7.5]
Modified Rankin score at admission	4 [3.5–5]	5 [4–5]
Modified Rankin score at discharge	3 [1.5–4]	4.5 [4–6]
Largest diameter of the infarct (mm)	–	45 [27.5–64]
TOAST classification	1: 4 (36%), 2: 1 (9%), 3: 6 (55%)	1: 2 (25%), 2: 5 (63%), 3: 1 (12%)

*Note*: Data are expressed as the median [IQR] or number (%).

Abbreviation: TT, thrombolytic therapy.

Eleven age‐matched controls with a comparable cardiovascular and metabolic risk profile provided peripheral blood samples on one occasion (age 76 [65–89] years, gender 4/7 male/female). Controls had a negative history of stroke and other neurological disorders. Patients and controls did not show clinical symptoms or laboratory findings suggestive of infection throughout their participation in the study. The study was approved by the local ethics committee of the recruiting institution. Informed consent was obtained from all participants, and the study adhered to the tenets of the most recent revision of the Declaration of Helsinki.

Note that 300 μl anticoagulated whole blood was incubated with the following conjugated anti‐human antibodies at 4°C following the manufacturers’ recommendations: CD15 FITC (clone C3D‐1; Dako, Glostrup, Denmark), CD45 PC7 (clone J33; Beckman Coulter, Miami, FL, USA), and CD64 PE (clone 10.1; Dako). After incubation, BD FACS Lysing Solution (Becton Dickinson, New Jersey, NJ, USA) was added for red cell lysis for 15 min at room temperature. Cells were washed and resuspended in phosphate buffered saline. A total of 500,000 cells were recorded from each sample using a Navios flow cytometer equipped with blue (488 nm) and red (638 nm) lasers (Beckman Coulter). The instrument was calibrated using Flow‐Set Pro and Flow‐Check Pro beads (Beckman Coulter). Data acquired from the measurements were evaluated with FlowJo software (version 10; Tree Star, Ashland, OR, USA). The populations of monocytes, granulocytes, and lymphocytes were gated based on CD45 positivity and side scatter characteristics . CD15+, CD15bright (highly positive), and CD64+ cells were gated within each population using histograms. In addition to the proportion of cells positive for a marker within the parent cell subset (%), mean fluorescence intensity (MFI) values were also determined. This reflects the abundance of the given marker on the cell surface within the population of cells expressing a given marker.

Data are expressed as the median [interquartile range]. Comparisons were made using Kruskal‒Wallis, Mann‒Whitney, and Wilcoxon signed rank tests. *p*‐Values less than .05 were considered significant. Statistics were calculated using GraphPad Prism 5 software (GraphPad, San Diego, CA, USA).

## 3 RESULTS

**FIGURE 1:**
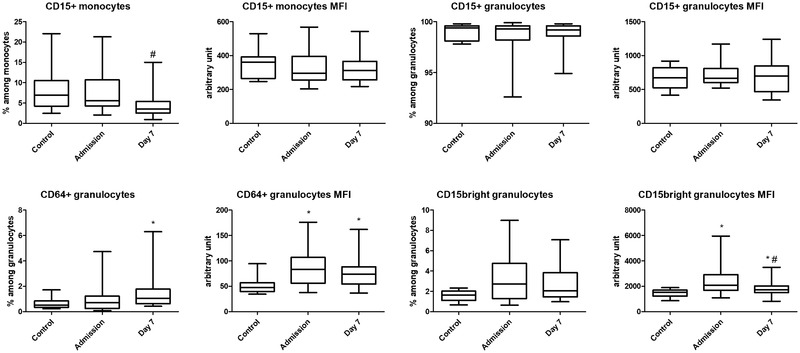
Comparisons of cell prevalence and mean fluorescence intensity (MFI) data between controls and acute ischemic stroke patients at admission and on day 7 following the insult. Data are presented as the median (horizontal line), interquartile range (box), and range (whiskers). *p < .05 vs. control, ^#^ *p* < .05 vs. admission

We first compared the investigated markers between controls and stroke patients at the two sampling time points. The proportion of monocytes was lower in stroke patients on admission than in controls but increased by day 7. The proportion of CD15+ monocytes was comparable with controls upon admission but decreased by day 7, resulting in a reduction in the absolute number of this cell subset by a week following the insult. Interestingly, the MFI of the CD15 marker on monocytes did not differ between the two time points (Figure 1). There was no difference in the proportion of CD64+ monocytes between the two time points. However, the MFI of CD64 on monocytes was higher in stroke patients at both time points than in controls.

The proportion of granulocytes and CD15+ granulocytes was comparable between controls and stroke patients at both time points. While the proportion of CD15bright granulocytes was also comparable, the MFI of CD15 on this subset was reduced in stroke patients by day 7 but was overall higher at both time points compared to controls. The proportion of CD64+ granulocytes was higher by a week after the insult in comparison to controls. The MFI of CD64 on granulocytes and lymphocytes was lower in controls than in stroke patients but comparable between the two time points (Figure 1). Of note, no further difference was observed in the expression of the examined markers on lymphocytes between controls and stroke patients or the two time points.

**FIGURE 2:**
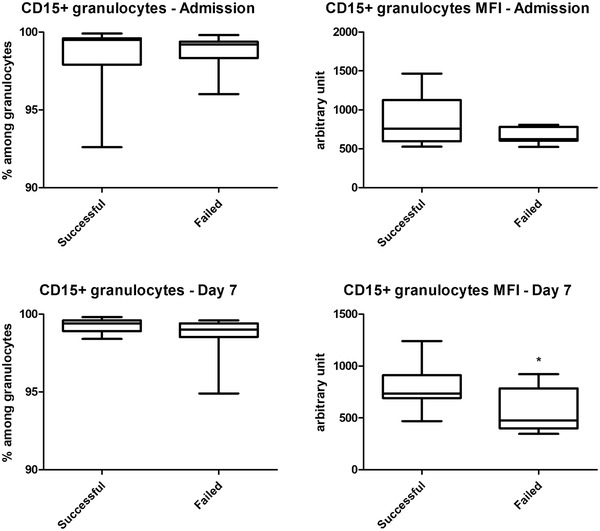
Comparisons of cell prevalence and mean fluorescence intensity (MFI) data between acute ischemic stroke patients with successful and failed thrombolytic therapy. Data are presented as the median (horizontal line), interquartile range (box), and range (whiskers). *p < .05 vs. successful

We next compared stroke patients with successful versus failed TT at the two sampling time points. The MFI of CD15 on granulocytes was lower in patients with failed TT on day 7 than in patients with successful TT. There was no difference in this marker on admission between the two patient subgroups (Figure 2). All other investigated markers were comparable between patients with successful and failed TT at both time points.

## 4 DISCUSSION

TT may not only reduce neurological impairment and complications associated with ischemia but may also have a positive effect on the evolving SIIS, thus improving the rates and outcome of poststroke infections. A better understanding of the dynamic changes in key markers of granulocyte function following stroke in association with TT therefore has important future diagnostic and therapeutic potential.

Although CD15 is primarily regarded as a granulocyte marker, it is also expressed on monocytes (Nakayama et al., 2001). Interestingly, the proportion of monocytes expressing this marker decreased by day 7 in stroke patients. The MFI of CD15 in granulocytes only showed stroke‐associated changes when examined within the CD15bright subset. This subset of high CD15 expression is often considered to correspond to the promyelocytic stage of neutrophil granulocyte differentiation (van Lochem et al., 2004). Within CD15bright granulocytes, the MFI of this marker showed a biphasic change, increasing with the onset of stroke but decreasing by day 7 (Figure 1). This decrease may reflect the evolving SIIS.

In our earlier study (Folyovich et al., 2014), we observed a similar biphasic change in the proportion of CD64+ granulocytes. Interestingly, we were unable to confirm this earlier finding in our present work, as the proportion of CD64+ granulocytes increased rather than decreased, while the MFI of this marker remained elevated on day 7 when compared to admission (Figure 1). This controversy may be related to the clinical characteristics of stroke patients participating in the two studies. In our present cohort, the majority (58%) of stroke patients had successful TT. In contrast, in our previous study (Folyovich et al., 2014), the proportion of patients with successful TT was much lower (9%), likely increasing the incidence and severity of SIIS in that specific cohort.

While CD64 expression on granulocytes did not differ between patients with successful versus failed TT, the MFI of CD15 on granulocytes showed a marked reduction in patients with failed TT 1 week after the insult (Figure 2). The associated reduced adhesional, phagocytic, and degranulation ability of granulocytes may contribute to a more pronounced immunosuppressive response, possibly putting these patients at a higher risk of poststroke infection‐associated complications. The exact molecular and cellular mechanisms governing these changes have yet to be determined.

While earlier studies suggest that TT reduces the inflammatory response following stroke, our current results indicate that it may also play a role in reversing SIIS at a later stage following the insult. Audebert et al. and later Ye et al. both reported that TT may inhibit the necrosis of brain tissue and thereby reduce the inflammatory response up to 5 days following the insult (Audebert et al., 2004; Ye et al., 2015). While reducing local inflammation, TT likely also influences the signaling mechanisms responsible for the onset of systemic SIIS (Santos Samary et al., 2016).

## 5 CONCLUSIONS

Our current results indicate that TT may not only acutely reduce the systemic inflammatory response following stroke but may also play a role in reversing SIIS at a later stage following the insult, reflected by the higher expression of the CD15 marker on granulocytes following successful TT. Our observation in human samples needs to be further confirmed in relevant animal models.

## CONFLICT OF INTEREST

The authors declare no conflict of interest.

### PEER REVIEW

The peer review history for this article is available at: https://publons.com/publon/10.1002/brb3.2732.

## Data Availability

The data that support the findings of this study are available from the corresponding author upon reasonable request.
